# Unpacking power dynamics in research and evaluation on social accountability for sexual and reproductive health and rights

**DOI:** 10.1186/s12939-021-01398-2

**Published:** 2021-02-06

**Authors:** Marta Schaaf, Suzanne Cant, Joanna Cordero, Sana Contractor, Etobssie Wako, Cicely Marston

**Affiliations:** 1 Independent Consultant, 357 Sixth Ave., NY 11215 Brooklyn, USA; 2 World Vision International, 39 Garden St, Blairgowrie, Victoria 3942 Australia; 3 Development and Research Training in Human Reproduction (HRP), Department of Sexual and Reproductive Health and Research, World Health Organization, 20 Avenue Appia, 1211 Geneva, Switzerland; 4 COPASAH Sexual and Reproductive Rights Hub, CHSJ, Basement of Young Women’s Hostel No 2, Avenue 21, G block, Saket, New Delhi, 110017 India; 5 CARE USA, 151 Ellis Street NE, Atlanta, GA USA; 6 Faculty of Public Health and Policy, London School of Hygiene & Tropical Medicine, London, WC1E 7HT UK

**Keywords:** Social accountability, Sexual and reproductive health and rights, Power, Measurement, Research

## Abstract

Over the past decade, social accountability for health has coalesced into a distinct field of research and practice. Whether explicitly stated or not, changed power relations are at the heart of what social accountability practitioners seek, particularly in the context of sexual and reproductive health. Yet, evaluations of social accountability programs frequently fail to assess important power dynamics. In this commentary, we argue that we must include an examination of power in research and evaluation of social accountability in sexual and reproductive health, and suggest ways to do this. The authors are part of a community of practice on measuring social accountability and health outcomes. We share key lessons from our efforts to conduct power sensitive research using different approaches and methods.

First, participatory research and evaluation approaches create space for program participants to engage actively in evaluations by defining success. Participation is also one of the key elements of feminist evaluation, which centers power relations rooted in gender. Participatory approaches can strengthen ‘traditional’ health evaluation approaches by ensuring that the changes assessed are meaningful to communities.

Fields from outside health offer approaches that help to describe and assess changes in power dynamics. For example, realist evaluation analyses the causal processes, or mechanisms, grounded in the interactions between social, political and other structures and human agency; programs try to influence these structures and/or human agency. Process tracing requires describing the mechanisms underlying change in power dymanics in a very detailed way, promoting insight into how changes in power relationships are related to the broader program.

Finally, case aggregation and comparison entail the aggregation of data from multiple cases to refine theories about when and how programs work. Case aggregation can allow for nuanced attention to context while still producing lessons that are applicable to inform programming more broadly.

We hope this brief discussion encourages other researchers and evaluators to share experiences of analysing power relations as part of evaluation of social accountability interventions for sexual and reproductive health so that together, we improve methodology in this crucial area.

## Background

Over the past decade, social accountability for health has coalesced into a distinct field of research and practice. Yet evaluations of social accountability programs frequently fail to assess important power dynamics. In this commentary, we argue that we must include an examination of power in research and evaluation of social accountability in sexual and reproductive health, and suggest ways to do this.

Power dynamics affect many areas of sexual and reproductive health, such as decision-making power within families, patient treatment in clinical settings, and professional relationships among health providers. The role of power in shaping health status and disparities, access to health care, the quality of care that communities receive, and social hierarchies (e.g., relating to caste, race, gender, ability, or class) has been theorised in many disciplines – ranging from social psychology to anthropology and political science [1, 2]. Failure to assess power dynamics can mean failure to identify crucial contextual characteristics that must change to improve sexual and reproductive health. Relevant characteristics include those that the program directly seeks to change, such as power relations between patients and health providers, or power dynamics that influence implementation and success of social accountability efforts more indirectly, such as government commitment to funding family planning programs, or societal gender norms. For example, one midwifery program failed in part because the community midwives could not undertake the required travel because of prevailing gender and class norms which prevented them moving around freely [3]. In that case, understanding wider social hierarchies was crucial to understanding programme failure.

Non-governmental organizations, international organizations, government agencies, and grassroots actors employ social accountability strategies to effect change in sexual and reproductive health. Common tactics include sharing data on health system performance, and community engagement and dialogue with decision-makers [4]. These tactics have been deployed to address a broad range of sexual and reproductive health priorities, such as ensuring contraceptives are available, ensuring clinic hours are convenient, respectful patient care, and reducing health provider absenteeism. However, community engagement and data sharing do not necessarily ensure that the intervention affects power dynamics to benefit communities. For this reason, evaluations should assess power dynamics, making explicit whether and how the intervention addresses power. Collective action has the potential to transform power relations, including relations within communities, between communities and health system actors, and within health systems [5]. Collective action (as opposed to isolated individual efforts) can generate countervailing power, which can foster change by, for example, impelling formalised sanctions processes, shaming health providers, or raising provider awareness of community dissatisfaction. Whether explicitly stated or not, changed power relations are at the heart of what social accountability practitioners seek, particularly in the context of sexual and reproductive health. For example, social accountability efforts have resulted in historically oppressed groups gaining greater voice in articulating maternal health priorities, local health providers no longer treating patients rudely with impunity, and health care workers at the bottom of the professional hierarchy being able to successfully negotiate to receive the supplies they need [6–8].

The authors of this commentary are part of the community of practice on measuring social accountability and health outcomes convened by the Department of Sexual and Reproductive Health and Research, World Health Organization. Measurement and evaluation of social accountability is an evolving area; this evolution relates to broader discussions within the social accountability field about the importance of the political and social setting; the degree and depth of change possible with locally bounded, time limited efforts; and the importance of understanding social accountability programs and tactics in the context of long-term, iterative social change strategies [9–11]. In a systematic review of methods to measure the impacts of social accountability on reproductive, maternal, newborn, child and adolescent health, presented to the community of practice in 2018, Marston et al. concluded that qualitative data are crucial to exposing mechanisms and processes of change, as well as to elucidating the broader social, political, and historical context [9]. The authors also found that no studies took an explicit systems approach and the analyses generally did not examine the extent to which programs influenced or were influenced by power dynamics within the health system and broader social structures [9].

Understanding whether, how, and at what levels power relations are shifted can help us to understand program success, and, particularly if evaluations are theory-based, build the evidence base in the field of social accountability [1, 9]. Recognizing that there are no ‘off the shelf’ solutions to very complex research and evaluation challenges, we share key lessons from our efforts to conduct power-sensitive research and evaluation using different methodological approaches. We seek dialogue with others who have used these approaches, as well as with those who have used alternative approaches, as we build the evidence base on how to assess power in research and evaluation on social accountability for sexual and reproductive health.

## Participatory research and evaluation

Participatory research and evaluation center power relations by asking program participants to definine the measures of success; these are often paired with participatory programming, wherein communities co-create the program goals, objectives, and activities.

Within sexual and reproductive health, participatory research and evaluation have been used to track and make sense of changes in health service utilization over time, jointly review outcomes, and identify corrective action. For example, a project team implementing a social accountability project aiming to improve the quality of care in services aimed at preventing mother-to-child transmission of HIV in Malawi developed indicators of care “in a consultative, participatory process … [where] participants discussed each indicator and agreed on a … score” [12]. These measures were then tracked to assess program success over time [12]. Participatory research and evaluation approaches can strengthen ‘traditional’ health evaluation approaches by ensuring that the changes assessed are meaningful to communities and that research design or evaluation is acceptable and feasible [13].

Participation is also one of the key elements of feminist evaluation, in which analysis centers power relations relating to gender. Feminist evaluation positions gender inequity as a social justice concern, and requires the evaluator to problematize gender relations, or to defamiliarize or question prevailing understandings and assumptions about gender relations [14]. This approach may be particularly apt for sexual and reproductive health, where gender relations of power play a key role in shaping health status and disparities, access to health services, and ability to make demands on the state at local and national level [11, 15, 16]. Many feminist evaluators draw on principles proposed by Sielbeck-Bowen et al. [17], which highlight the structured and systemic nature of gender inequality and discrimination, the inherent politics of evaluation, and the recognition of multiple perspectives on what constitutes useful knowledge.

Hay [18] argues that feminist approaches to evaluation must inform our understanding of programs that seek to shift gender relations of power, such as efforts to enhance the voice of historically oppressed women, or to make sexual and reproductive health services more accessible and acceptable to them. For example, an evaluation of an intervention to improve adolescent girls’ access to sexual and reproductive health services in Uttar Pradesh, India, used feminist evaluation to assess the gender empowerment potential of the intervention through analysis of personal transformations among the girls themselves, the fostering of leaders and advocates within the girls’ groups, movement of the primary beneficiaries from being passive recipients to active citizens, and steps toward transforming community norms towards gender equality [19].

## Realist research and evaluation and process tracing

Power relations are inherently difficult to describe, and changes in power relations are difficult to assess. Approaches and methods developed outside of the health field that are sensitive to power relations are increasingly used in health to research and evalute programs, including social accountability for sexual and reproductive health. Two key approaches include realist evaluation and process tracing.

Realist approaches seek to explain what works for whom, why, and in what circumstances. To do this, evaluators analyse the causal processes, or mechanisms, grounded in the interactions between social, political and other structures and human agency; programs try to influence these structures and/or human agency. Mechanisms are triggered in certain contextual conditions and not in others, drawing attention to the importance of context and its effect on agency. Realist evaluation and research can help practitioners to understand the conditions in which interventions are likely to be successful [20].

The concepts of structure and agency here can be related to power in that hierarchies of power constitute a structure, limiting agency, such as the intersectional influence of gender norms and social hierarchies on disabled women’s ability to obtain contraception from their local health facility. Realist evaluation has been used to analyze how different actors interact in the context of broader power dynamics. For example, in one study, researchers assessed shifts in power within communities and between communities and their local government [21]. When people discussed their health services in groups of peers, they could better share their opinions than in mixed groups. These opinions were then shared with government officials in broader town hall meetings. The realist evaluators found that the combination of the intervention’s activities created a ‘legitimating’ and ‘authorizing’ effect for both service users and providers to access and influence higher levels of government [21].

Process tracing can help to refine program theory by gathering clues to assess the likelihood that a given outcome was due to a putative cause. In this approach, the mechanisms underlying change in power dymanics are analysed and described in a very detailed way, promoting insight into how changes in power relationships are related to the broader program.

Process tracing has been used to identify when and where community members are able to lobby regional or district authorities for changes rather than only local providers, as well as analysing power dynamics among different groups in community meetings [8, 22]. Process tracing can be particularly useful in assessing variations in policy implementation and the drivers for these differences, such as a study conducted by Ruibal that describes the political determinants of differing levels of compliance with abortion law in Argentina [23].

## Case aggregation and comparison

As well as examining specific programmes, case studies can be combined to provide further insights. Researchers can employ a range of approaches and methods, from inductive grounded theory to deductive hypothesis testing, to aggregate data from multiple cases to develop and/or refine theories about when and how programs work. Case aggregation can allow for nuanced attention to context while still producing lessons that are broadly applicable to inform programming. For example, Lodenstein et al. collated cases documented in peer-reviewed and “grey” literature, such as project reports, to describe key mechanisms of health provider responsiveness to programs seeking to promote social accountability for maternal health, finding that responsiveness was dependent in part on health providers’ perceptions of the legitimacy of the groups making demands, and that social accountability efforts seemingly effected more transformative change in countries that were undergoing broader democratization [5].

Hernandez et al. use qualitative comparative analysis to identify and compare outcomes of citizen-led initiatives for the right to health of indigenous populations, finding different pathways of network building, iterative cycles of collective action, and the successful use of both constructive and adversarial engagement strategies [24]. Depending on the area of interest, aggregation and comparison might be most productive across meaningfully bounded categories, such as sub-nationally (i.e. comparing and aggregating within the same country), in situations with a similar sexual and reproductive health context (e.g., where early marriage is criminalized), or with a particular focus on program mechanisms (e.g., instigating punishment of health providers). Subnational case aggregation and comparison can help researchers to identify manifestations of power asymmetries, such as the gender composition of the local council, the political party in power at the local level, or the racial/ethnic composition of the communities addressed, that help to explain differences in program outcomes.

## Centering power in research

Many of the methodological approaches we have described above rely primarily on qualitative tools for data collection. However, it is important to note that practitioners and researchers have developed quantitative tools to assess individual or group empowerment; these may be used in a quasi experimental design to detect change over time. Measures assess phenomena such as group efficacy, agency, and action among women and health providers in the context of reproductive and maternal health social accountability programming [25–27]. Regardless of the tools used, explicit consideration of power dynamics is essential to understanding the contextual factors that shape the implementation of social accountability programmes, as well as understanding their impact.

In addition to its instrumental utility in research and evaluation, attention to power within the research process can help the research itself to be transformative. Participatory approaches may be explicitly oriented towards overturning long-standing power dynamics among researchers, implementers, and community members [28]. Such methods can deepen community participation in program evaluation, create pathways for collective learning, and position communities as knowers and partners [28]. The decolonizing global health movement points to other elements of the research process – such as the disciplines, regions, experiences, and social locations represented by research team members – as features that can perpetuate or alter prevailing power dynamics [29]. Diverse teams and participatory approaches can be integrated into all the approaches and methods described here.

We hope this discussion encourages other researchers and evaluators to share experiences of analysing power relations as part of evaluation of social accountability interventions for sexual and reproductive health so that together, we improve methodology in this crucial area. Failing to engage questions of power in sexual and reproductive health interventions – which can be highly politicised [30, 31] – risks undermining attempts to improve health and rights by locating problems solely in individual behaviour or characteristics that need to be fixed, glossing over the impact of political structures and entrenched social hierarchies. While these structures and hierarchies are resistant to change, they are at the heart of what must change to meaningfully improve sexual and reproductive health.

## Data Availability

Data sharing not applicable to this article as no datasets were generated or analysed.
